# Supplementary material for: Anti-AMPA Receptor Autoantibodies Reduce Excitatory Currents in Rat Hippocampal Neurons
Source: Pharmaceuticals (Basel). 2023 Jan 4;16(1):77. doi: 10.3390/ph16010077 (PMC9864520; doi:10.3390/ph16010077)
Supplement: Supplementary file 1 [file pharmaceuticals-16-00077-s001.zip › pharmaceuticals-2085300-supplementary.pdf]

Supplemental Figure S1

A

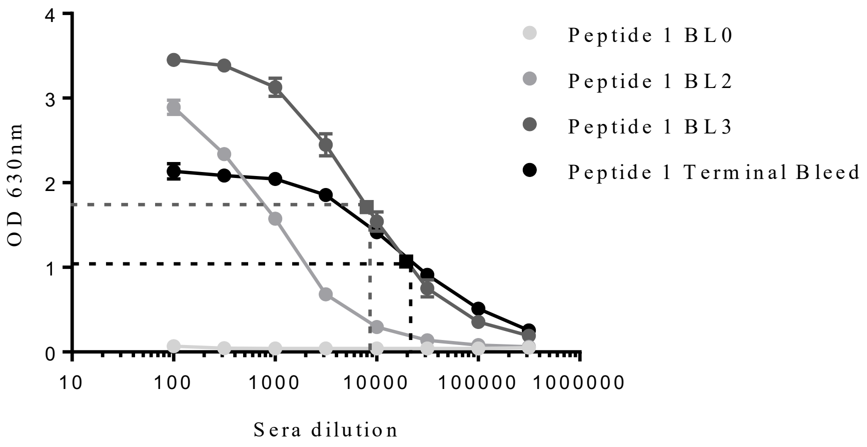

B

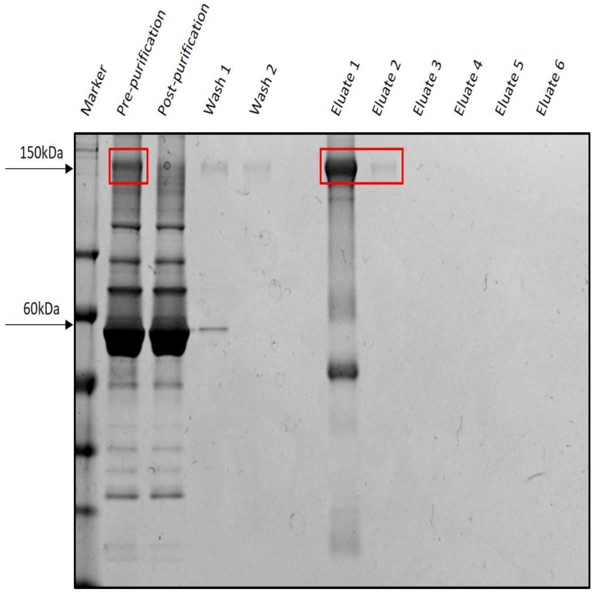

C

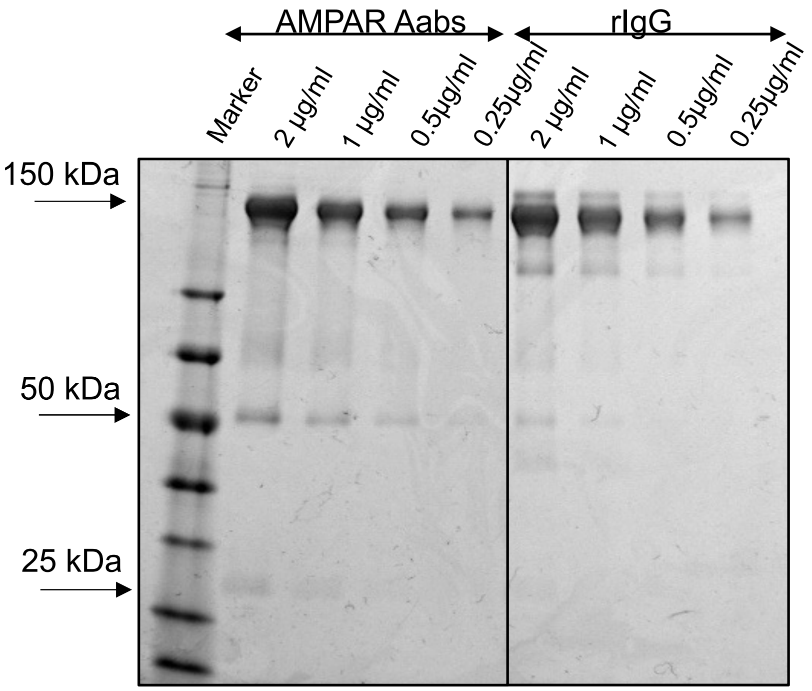

Supplemental Figure S2

A **commercial anti-AMPAR Ab**

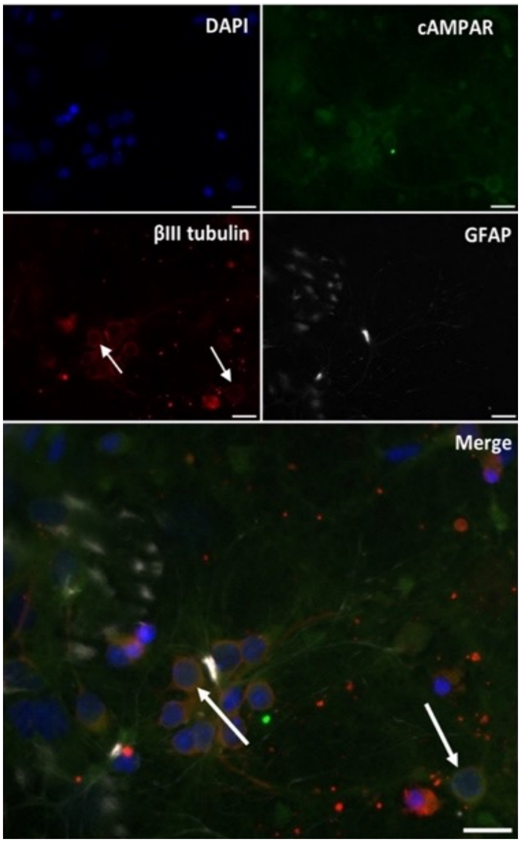

B **Secondary Ab only**

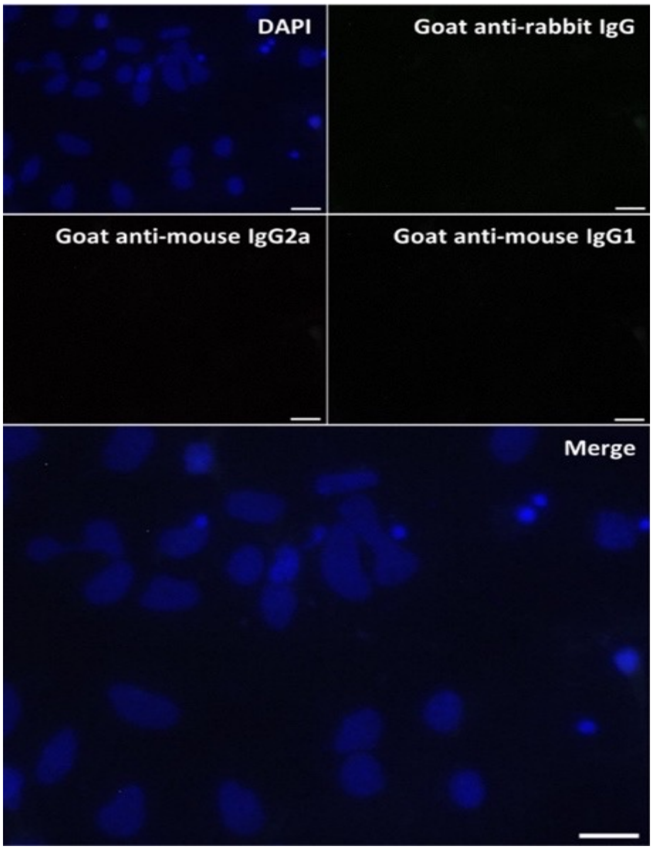

C **rigG**

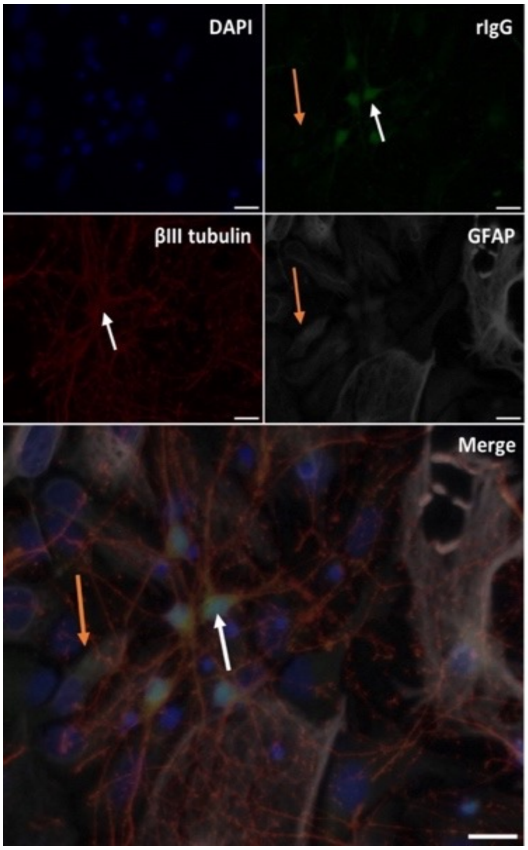

Supplemental Figure S3

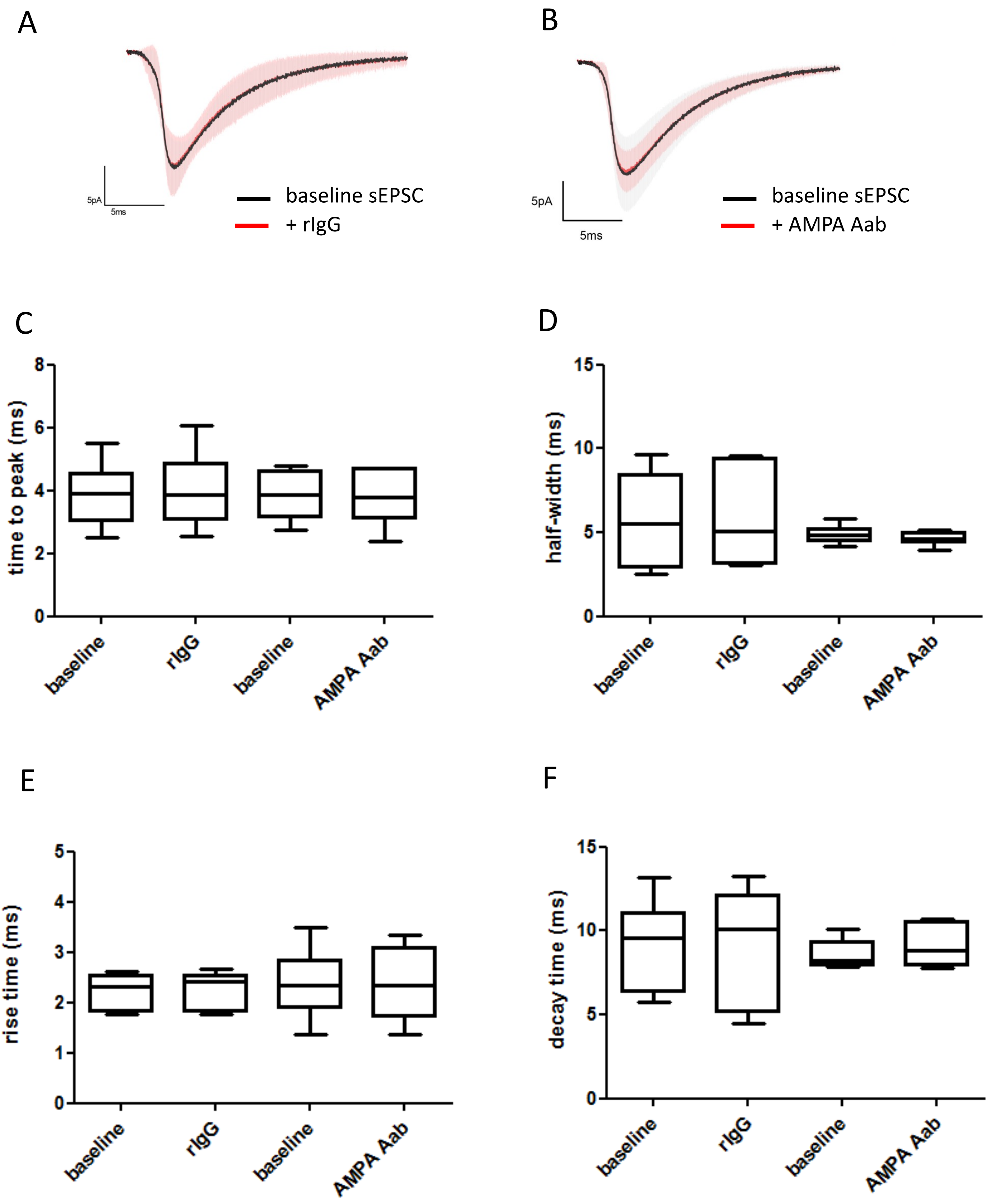

Supplemental Figure S1. Rabbit anti-AMPAR GluR3B immunogenicity response.

A) Pre-immunisation bleed (BL0), bleed 2 (BL2), 3 (BL3) and terminal bleed responses to AMPAR Glur3B peptide used for immunisations. An increase in response to the peptide was observed with each immunisation boost. Dotted lines show half maximum values for BL3 and Terminal bleed. N=3 technical replicates. B) Protein A purification of anti-AMPAR Aabs. SDS-PAGE analysis of rabbit serum purified by protein A-sepharose resin. Analysis of all fractions revealed a specific band at 150 kDa in pre-purification sample and eluates 1-2 (as indicated by the red boxes), which is not present in the post-purification sample. Figure 1C) Protein A purification of anti-AMPAR Aabs. SDS-PAGE analysis of rabbit serum purified by protein A-sepharose resin compared with control rabbit IgG of known concentrations. Analysis revealed a single band at the expected size of 150 kDa across all dilutions for both AMPAR Aabs and the control rIgG.

Supplemental Figure S2. Immunocytochemical staining of fixed primary cortical neurons (DIV5-10).

(A) Cells were stained with commercial anti-AMPAR antibodies (cAMPAR; green, 1:100), βIII tubulin, GFAP and a nuclear stain DAPI. Clear staining was seen with cAMPAR (green) as indicated by the white arrows. (B) Cells were stained with secondary antibodies only. Goat anti-rabbit IgG (green), goat anti-mouse IgG2a (red) and goat anti-mouse IgG1 (white) and DAPI. No labelling was detected in any of these channels. (C) Cells were stained with rIgG (green, 1:100), βIII tubulin; a neuronal marker (red, 1:500), GFAP; an astrocyte marker (white, 1:400) and a nuclear stain (DAPI: blue, 1:10,000). Faint staining was detected with rIgG (green), which co-localised with βIII tubulin-stained cells (as indicated by white arrows), as well as cells labelled by GFAP (orange arrows). Scale = 20μm. Representative images selected from n=3 technical replicates.

Supplemental Figure S3. Biophysical properties of sEPSCs.

sEPSCs under baseline conditions and in the presence of anti-AMPA Aabs (1:1000) and rIgG (1:1000) were fitted with a template search function (Clampfit, Molecular Devices, UK) to derive the parameters shown. (A) Overlay of sEPSPs under baseline and subsequent application of rIgG. Baseline parameters of (C) time to peak, (D) half-width, (E) rise time and (F) decay time were unaffected by anti-AMPA Aabs and rIgG; there was no difference in any parameter between anti-AMPA Aabs and rIgG. Analysis based on events in n=6 independent experiments/ condition; throughout; $P>0.05$ (one-way ANOVA).
